# Tremor after long term lithium treatment; is it cortical myoclonus?

**DOI:** 10.1186/s40673-019-0100-y

**Published:** 2019-05-22

**Authors:** Ptolemaios Georgios Sarrigiannis, Panagiotis Zis, Zoe Charlotte Unwin, Daniel J. Blackburn, Nigel Hoggard, Yifan Zhao, Stephen A. Billings, Aijaz A. Khan, John Yianni, Marios Hadjivassiliou

**Affiliations:** 1 Department of Clinical Neurophysiology, Sheffield Teaching Hospitals NHS Foundation Trust, Royal Hallamshire Hospital, Floor N., Sheffield, UK; 2 Department of Neurology, Sheffield Teaching Hospitals NHS Foundation Trust, Sheffield, UK; 3 Department of Neuroradiology, Sheffield Teaching Hospitals NHS Foundation Trust, Sheffield, UK; 4 Through-life Engineering Services Centre, Cranfield University, Bedford, MK43 0AL UK; 5 Department of Automatic Control and Systems Engineering, University of Sheffield, S1 3JD, Sheffield, UK; 6 Department of Neurosurgery, Sheffield Teaching Hospitals NHS Foundation Trust, Sheffield, UK

**Keywords:** Lithium, Cerebellar ataxia, Cortical myoclonus, Gluten sensitivity, JLA, MRS

## Abstract

**Introduction:**

Tremor is a common side effect of treatment with lithium. Its characteristics can vary and when less rhythmical, distinction from myoclonus can be difficult.

**Methods:**

We identified 8 patients on long-term treatment with lithium that developed upper limb tremor. All patients were assessed clinically and electrophysiologically, with jerk-locked averaging (JLA) and cross-correlation (CC) analysis, and five of them underwent brain MRI examination including spectroscopy (MRS) of the cerebellum.

**Results:**

Seven patients (6 female) had action and postural myoclonus and one a regular postural and kinetic tremor that persisted at rest. Mean age at presentation was 58 years (range 42–77) after lengthy exposure to lithium (range 7–40 years). During routine monitoring all patients had lithium levels within the recommended therapeutic range (0.4-1 mmol/l). There was clinical and/or radiological evidence (on cerebellar MRS) of cerebellar dysfunction in 6 patients. JLA and/or CC suggested a cortical generator of the myoclonus in seven patients. All seven were on antidepressants and three additionally on neuroleptics, four of them had gluten sensitivity and two reported alcohol abuse.

**Conclusions:**

A synergistic effect of different factors appears to be contributing to the development of cortical myoclonus after chronic exposure to lithium. We hypothesise that the cerebellum is involved in the generation of cortical myoclonus in these cases and factors aetiologically linked to cerebellar pathology like gluten sensitivity and alcohol abuse may play a role in the development of myoclonus. Despite the very limited evidence in the literature, lithium induced cortical myoclonus may not be so rare.

**Electronic supplementary material:**

The online version of this article (10.1186/s40673-019-0100-y) contains supplementary material, which is available to authorized users.

## Introduction

Lithium induced tremor is a common side effect and can occur both early and after many years of exposure. When manifesting early it usually consists of a fine tremor that can occur at rest, be postural and/or kinetic, often unrelated to blood levels [1–10]. Myoclonus is another movement disorder manifesting during treatment with lithium [11]. When myoclonus presents as a tremor it can be challenging to clinically recognise, particularly when not stimulus sensitive. Typically tremor consists of regular (frequency and amplitude) stereotyped repetitive movements [12]. Shibasaki and Hallett make the argument that electrophysiology is essential in the diagnosis of myoclonus because “… myoclonus is difficult to distinguish from other involuntary movements such as tremor, chorea, and dystonia” and “… cortical myoclonus frequently appears to be rhythmic” [13]. Although myoclonic movements are usually assumed to be “sudden, brief and shock like” some forms of myoclonus may fit into the broad definition of tremor [14]. The importance of electrophysiology in distinguishing tremor from myoclonus is also highlighted by Caviness and Brown [15]. As distinction between tremor and myoclonus can be difficult in the clinical setting, polygraphy recordings and electrophysiological analysis that are only available in specialised centres, can assist in the classification [16] and identification of the anatomical generator of the myoclonus.

In cortical myoclonus, a paroxysmal depolarisation shift (PDS) involving the cerebral motor cortex, drives the corticospinal volley that generates the myoclonic discharge [13, 17]. The cortical spikey EEG correlate of the PDS varies in morphology and can be negative if the PDS is widely distributed across the neocortex or positive if restricted in the deep layers of the motor cortex [18]. On the other hand, centrally driven tremors are characterised by a widely distributed tremorogenic network and quantitative EEG/EMG analysis with jerk-locked averaging (JLA) and/or cross-correlation (CC) is not expected to produce sharp cortical transients before the tremulous EMG discharges.

In this work, we discuss our experience with 8 consecutive patients referred for electrophysiological assessment of their tremulous motor manifestations whilst on long-term treatment with lithium.

## Methods

### Standard protocol approvals, registrations and patient consents

This is an observational study. Five of the patients included in this work have been referred to the Sheffield Ataxia Centre, Royal Hallamshire Hospital, Sheffield, UK because of balance problems while the remaining three cases were seen at a general neurology clinic primarily because of upper limb tremor. The South Yorkshire Research Ethics Committee has confirmed that no ethical approval is required given that all investigations were clinically indicated and did not form part of a research study. The specialised electrophysiological recordings and quantitative analysis are routinely performed at the Department of Clinical Neurophysiology at the Royal Hallamshire Hospital (Sheffield, UK) and all patients have provided written consent.

### Case selection

Eight consecutive cases on chronic treatment with Lithium referred to the department of Clinical Neurophysiology at the Royal Hallamshire Hospital (Sheffield, UK) for analysis of their tremulous manifestations were identified for this report.

### Neuroimaging

Five of the patients (Table 1, cases 1–5) underwent brain MRI and magnetic resonance spectroscopy (MRS) of the vermis and the hemispheres [19]. This latter technique is very useful in providing evidence of cerebellar dysfunction independently from the presence of atrophy [20–22]. The N-acetyl-aspartate/creatine, NAA/Cr, ratio is used in our institution and the Standardized normal values are NAA/Cr > 0.95 in the vermis and > 1.00 in the hemispheres [19].

**Table 1:** Electroclinical and neuroimaging findings

Case no	Age at onset (Gender)	Years on lithium	Gluten sensitivity	Cerebellar MRS	JLA & CC^c^	SEPs	Postural tremor^e^ frequency	Psychotropic medication daily doses
*1*	*77y, F*	*40y*	*–*	*Reduced NAA/Cr* ^a^ vermis 0.91 hemispheres 0.78	*Action and stimulus reflex cortical myoclonus*	*“Giant” & C-reflexes* ^b^	*14 Hz*	*Lithium* ^*d*^ *400 mg* *Mirtazapine 45 mg*
*2*	*63y, F*	*10y*	*+ IgG*	*Reduced NAA/Cr* *vermis 0.87* *hemispheres 1.04*	*Cortical myoclonus*	*normal*	*10 Hz*	*Lithium 400 mg* *Mirtazapine 45 mg* *Sertraline 200 mg*
*3*	*57y, M*	*7y*	*+ IgG*	*Reduced NAA/Cr* *vermis 0.79* *hemispheres 1.04*	*Cortical myoclonus*	*normal*	*10 Hz*	*Lithium 408 mg* *Venlafaxine 225 mg*
4	*47y, F*	*10y*	*–*	*Reduced NAA/Cr* *vermis 0.75* *hemispheres 0.8*	*Cortical myoclonus*	*normal*	*11 Hz*	*Lithium 800 mg* *imipramine 100 mg* *Olanzapine 25 mg*
*5*	*55y, F*	*30y*	*+ IgG*	*Reduced NAA/Cr* *vermis 0.89* *hemispheres 0.85*	*Cortical myoclonus*	*normal*	*10 Hz and first harmonic at 20 Hz*	*Lithium 675 mg* *Citalopram 40 mg* *Quetiapine 100 mg*
*6*	*70y, F*	*20y*	*+ IgG*	*ND*	*Cortical myoclonus*	*normal*	*8 Hz*	*Lithium 400 mg* *fluoxetine 40 mg*
*7*	*42y, F*	*10y*	*–*	*ND*	*Cortical myoclonus*	*normal*	*13 Hz*	*Lithium 1000 mg* *Quetiapine 650 mg* *Sertraline 100 mg*
*8*	*66y, M*	*29y*	*–*	*ND*	*Central tremor*	*normal*	*6 Hz and first harmonic at 12 Hz*	*Lithium 800 mg* *Flupenthixole 0.5 mg*

*MRS* Magnetic resonance spectroscopy, *ND* not done, *SEP* somatosensory evoked potentials

^a^The NAA/Cr, N-acetyl-aspartate/creatine. This was measured in the vermis and cerebellar hemispheres (normal values Vermis > 0.95, hemispheres > 1.00

**Fig. 1:**
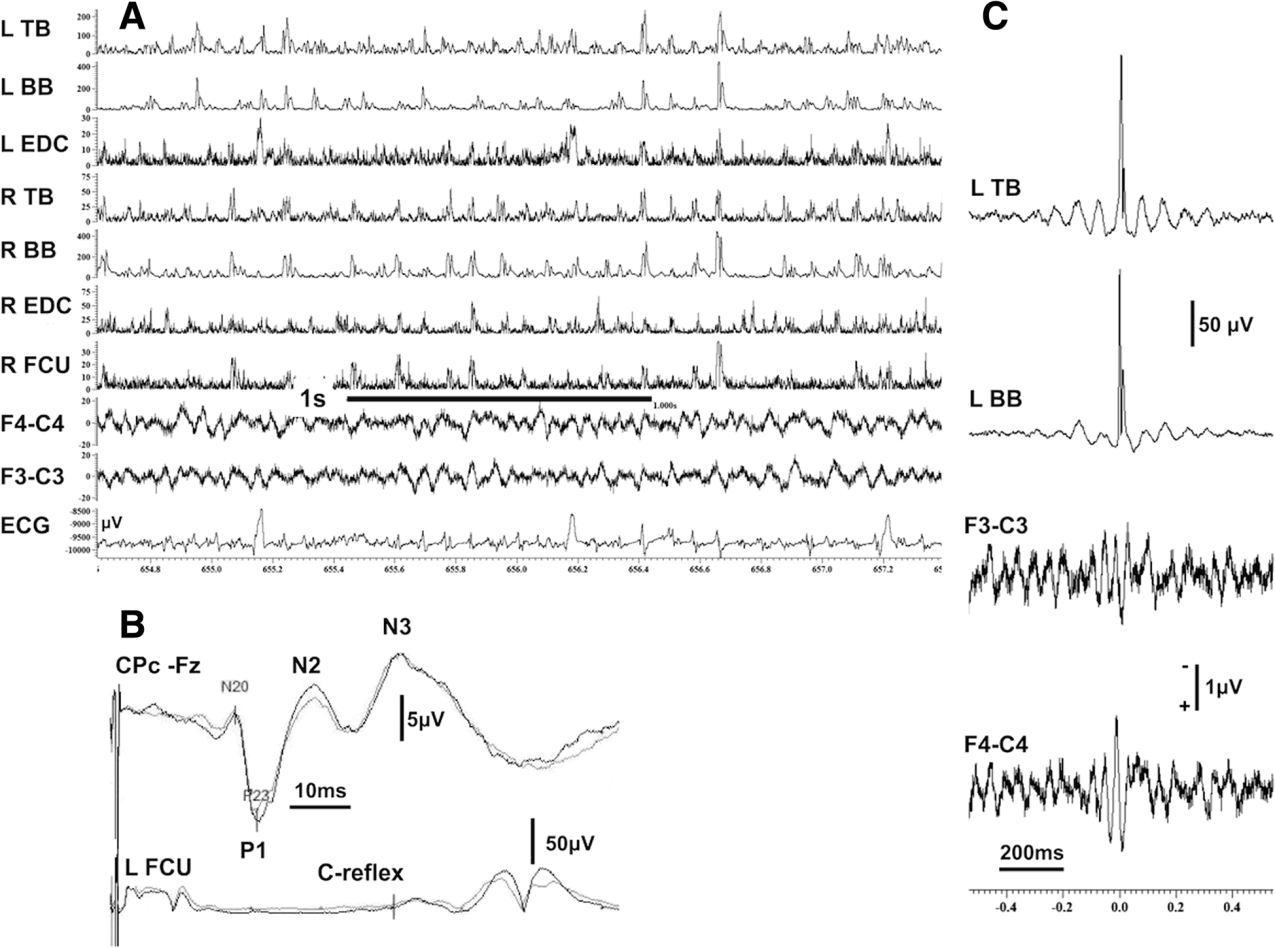
**a** EEG/EMG polygraphy showing the irregular myoclonic tremor. The most prominent myoclonic jerks involve synchronously both upper limbs and proximal and distal muscles. **b** SEPs from the same patient showing “Giant” cortical potentials and C-reflexes indicating cortical hyperexcitability and stimulus sensitive myoclonus. **c** JLA for the same patient from the left upper arm. There is a biphasic, positive/negative, spiky transient preceding by few milliseconds the rectified and averaged myoclonic EMG discharges (960 sweeps were averaged). The cortico-muscular latencies are shown at a slower sweep time in the relevant Additional file 1. APB = abductor pollicis brevis, BB=Biceps brachii, FCU = flexor carpi ulnaris, EDC = extensor digitorum communis, JLA = jerk-locked averaging, SEP = somatosensory evoked potentials, TB = triceps brachii

^b^The P_1_N_2_ and the P1N3 components were enlarged, above 14 μV (Fig. 1b)

**Fig. 2:**
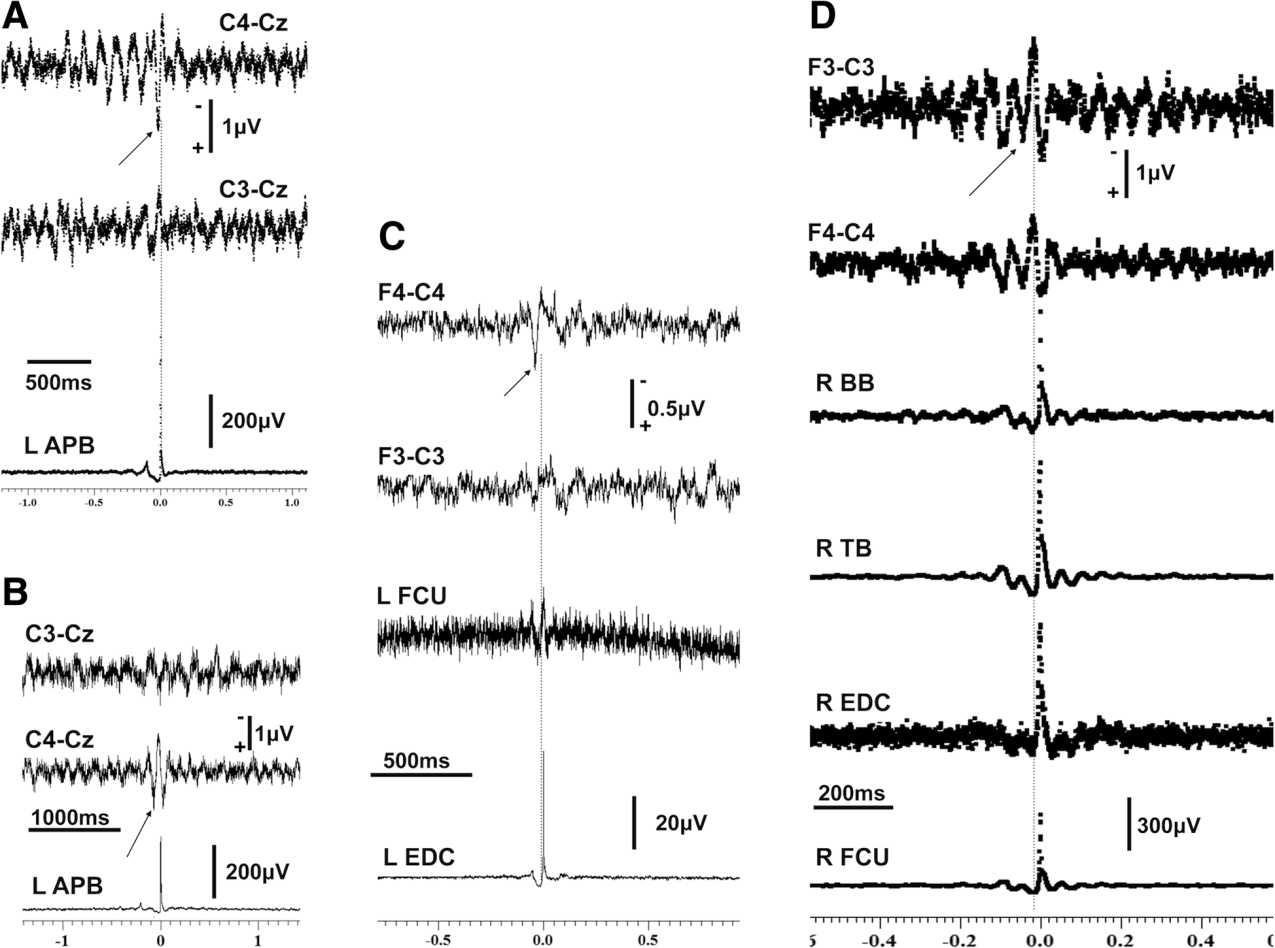
Results of JLA for 4 of the cases that were gluten positive (**a**), (**b**), (**c**), (**d**). A sharp biphasic transient appears in the contralateral central (**a** and **b**) and in the contralateral frontocentral EEG channel preceding the onset of the averaged EMG discharges in (**c** and **d**). The cortico-muscular latencies are shown at a slower sweep time in the relevant Additional file 1. APB = abductor pollicis brevis, BB=Biceps brachii, FCU = flexor carpi ulnaris, EDC = extensor digitorum communis, JLA = jerk-locked averaging, SEP = somatosensory evoked potentials, TB = triceps brachii

**Fig. 3:**
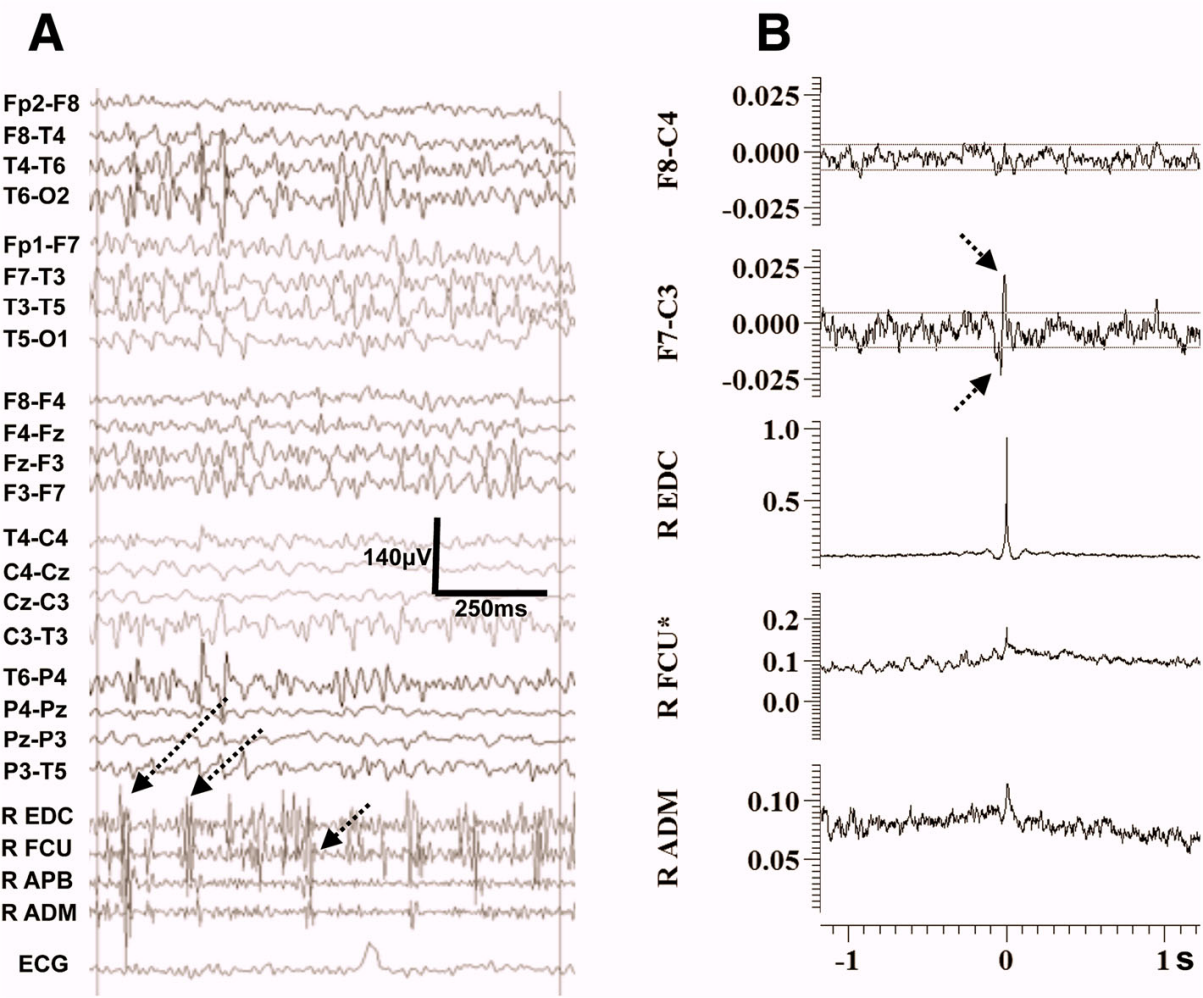
Electrophysiological findings from case 6. **a** EEG/EMG polygraphy showing on a 1 s epoch the irregular myoclonic jerks, consisting of small duration EMG discharges < 50 ms, demonstrating frequent co-activation of forearm antagonists with occasional simultaneous co-contraction of intrinsic hand muscles. **b** Averaged data obtained with the technique of CC with the tremorogenic activity from the R EDC used as reference (note the auto-correlation for the R EDC is 1). Spiky biphasic transients appear in the contralateral frontocentral EEG channel preceding the peak of the autocorrelation findings from the EDC by a few milliseconds. Two minutes of this jerky activity were used on the analysis. Please note the dotted lines indicate the 95% confidence interval. The cortico-muscular latencies are shown at a slower sweep time in the relevant Additional file 1. ADM = abductor digiti minimi**,** APB = abductor pollicis brevis, FCU = flexor carpi ulnaris, EDC = extensor digitorum communis

**Fig. 4:**
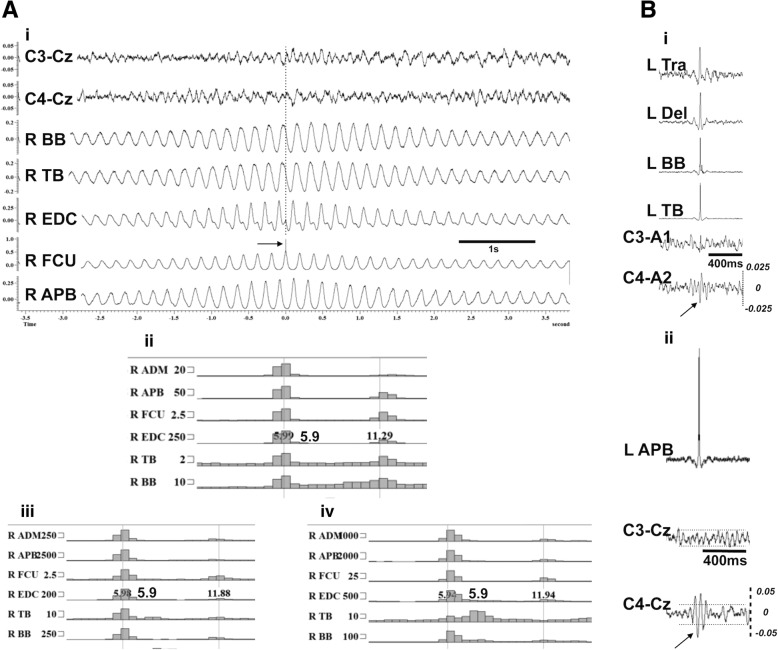
**a** CC analysis of the right arm tremor polygraphy recordings from case 8. This reveals typical sinusoidal oscillations at a frequency of around 6 Hz with a mainly out of phase relationship between antagonist muscles in the forearm (FCU/EDC) and out of phase activation between proximal and distal muscles. The right FCU was used as reference (i). FFT analysis (blocks of 4096 data points at a resolution of 0.5 Hz) was implemented on the EMG recordings while maintaining antigravity posture; it reveals a tremor at 5.9 Hz with its first harmonic (ii). Same analysis after loading the hand initially with 0.5Kg (iii) and then with 1 kg (iv) does not show any modification to the peak frequency of the tremor, as expected for a centrally driven tremor. The electrophysiological findings from Case 8, shown in (**a**) versus another patient with cortical myoclonic tremor, case 7 shown in (**b**). The arrows show the contralateral to EMG biphasic cortical transients on the averaged EEG data in the central areas. The EMG polygraphy shows co-activation of proximal and distal muscles (i). The horizontal dotted lines in the central derivations represent the 95% confidence interval; the biphasic cortical transient preceding by few milliseconds the peak of the autocorrelation from the EDC is outside the 95% margins (ii). The cortico-muscular latencies for case 7 are shown at a slower sweep time in the relevant Additional file 1. Noticeably, the cross-correlation analysis in **a** and **b** show the regular sinusoidal oscillations in the former, typically seen in tremors, and in the latter, the synchronous proximal and distal activation that can be expected in cortically driven myoclonus. ADM = abductor digiti minimi, APB = abductor pollicis brevis, BB = biceps brachii, Del = deltoid, FCU = flexor carpi ulnaris, EDC = extensor digitorum communis, TB = triceps brachii, Tra = trapezius

^c^JLA, jerk locked averaging and CC, cross correlation analysis were applied in cases 1–8, results are shown in Figs. 1, 2, 3 and 4

^d^All patients had lithium serum levels within the normal range: 0.4-1 mmol/L and all received a dose below 1200 mg/die of lithium carbonate or equivalent dose of lithium citrate (liquid preparation)

^e^Cases 1 to 7 presented with a quasi-regular tremor while case 8 was rhythmical. Frequencies were estimated with the in-built FFT function in spike 2 software on 20 s epochs of EMG recordings during upper limb maintenance of anti-gravity posture

### Electrophysiological assessment

All patients underwent electroencephalographic (EEG) and multichannel surface electromyography (EMG) polygraphy recordings to assess their upper limb tremulous activity. When rhythmical sinusoidal oscillations were seen (easily appreciated from appropriately placed hand accelerometers) then weight loading was used to identify a load-invariant EMG peak that suggests a central oscillator. The multichannel Natus Quantum amplifier (Optima Medical Ltd) at a sampling rate of 2048 Hz was used for all EEG/EMG polygraphy examinations (analogue bandwidth 0.01–680 Hz). Subsequently, data was exported for quantitative analysis on spike 2 (version 8.12) software (CED Ltd). Fast fourier transform (FFT) analysis was performed in one patient (case 8) before and after weight loading at the level of the wrist, to distinguish between peripheral and central components of the tremorogenic network [23]. The techniques of jerk-locked averaging (JLA) and cross-correlation (CC) analysis were applied in all cases. The in-built Spike 2 software functions for data averaging and correlation analysis, the latter averaging technique appears in previous publications as cumulant density [24, 25], were used. The technique of JLA was applied based on previous reports [13, 26–28] and EMG channels were rectified and direct current (DC) removed (time constant 0.05) to identify a clear onset of the EMG discharges. EEG data was not filtered. This enabled us to be able to record slow pre-movement cortical potentials that can be seen during participation of voluntary control systems in movement generation [27]. Events were marked from small duration (< 75 ms) myoclonic jerks, captured from the surface EMG electrodes. Several hundred to several thousand events were used for data analysis for each patient. Both JLA and CC, another data averaging [29] method, where applied. Somatosensory evoked potentials (SEP) recordings were made according to recent recommendations [30] and the possibility of cortical reflexes (C-reflexes) was examined with surface EMG electrodes in the leg and arm areas with the methodology and normal values described in previous work [31].

## Results

The demographics, age at presentation, years on lithium during onset of neurological manifestations, gluten sensitivity serology, MRS, electrophysiological findings and psychotropic medications with daily doses are described in Table 1.

On clinical examination, four patients (cases 1–3 and 5) had ataxia on finger-nose and heel to sheen examination, evidence of gait ataxia and an irregular upper limb postural and action tremor that tended to persist at rest. The other patient had similar jerky upper limb tremor, gaze evoked nystagmus, brisk reflexes and gait ataxia (case 4). For the remaining three patients (cases 6–8), upper limb jerky postural/action tremor, was the main symptom at the time of referral for a neurological opinion. Neurological examination revealed gait ataxia in case 7 who also had occasional jerks of the right leg. Case 8 had a family history of tremor (mother and three of his sisters were affected). Two of the eight patients (case 2 and 3) had a history of excessive alcohol intake and four had positive antigliadin antibodies in keeping with gluten sensitivity. Case 7 had previous gastric bypass surgery and two (case 3 and 7) were on treatment with thyroxine. Case 5 had a past history of thyrotoxicosis treated with carbimazole but was euthyroid at presentation.

None of the patients underwent any alterations of their lithium dose prior to the occurrence of the tremor and all had serum lithium levels consistently within the recommended therapeutic range (0.4-1 mmol/l) with no past history of lithium intoxication. All eight patients received various antidepressants (tricyclic antidepressants (TCA), imipramine; selective serotonin reuptake inhibitors (SSRIs), sertraline, citalopram, fluoxetine; noradrenergic and specific serotonergic antidepressant (NaSSA), mirtazapine; serotonin-norepinephrine reuptake inhibitor (SNRI), venlafaxine), and four received antipsychotic medication (typical antipsychotics, flupenthixole; atypical antipsychotics, quetiapine and olanzapine).

Radiologically, cerebellar atrophy was found on MRI in two cases, moderate (case 4) and mild (case 5), while five out of 7 had abnormal MRS with low NAA/Cr.

On electrophysiological examination, in five of the eight patients (cases 1–5) the EEG showed background rhythms lying within normal limits with no clear focal or epileptiform discharges. The EEG in case 6 was remarkable for occasional sharp/spike and slow wave discharges with a right frontotemporal and parietal emphasis. A single spike/slow wave discharge occurred in the left frontotemporal region on the record of case 7 and some non-specific bursts of slow wave activity, mainly theta, were seen on the recording from case 8. The background EEG of these latter two patients was lying within normal limits. There were no electro-clinical associations in any of the raw EEG/EMG polygraphy recordings. Results from the quantitative analysis of the data with JLA and CC showed evidence of cortical reflex myoclonus for case 1, with enlarged SEPs (Fig. 1) and of cortical myoclonus in cases 2–7 (Figs. 2, 3 and 4b, i and ii). The technique of CC, a linear averaging method that can be used for quantitative EEG/EMG data analysis, can be particularly useful when the differential diagnostic dilemma between tremor and myoclonus arises. Results from CC analysis for the only subject (case 8) with a rather sinusoidal tremor, that emerged after many years on treatment with lithium, and a strong family history of essential tremor are shown in Fig. 4 where the typical sinusoidal morphology of the oscillations, an expression of the relatively regular amplitude and frequency of the tremulous movements can be easily appreciated. Additionally, the averaged EMG polygraphy data exhibit variable out of phase relationships between proximal and distal muscles despite some pairs of antagonists show relatively in phase activation (Fig. 4, i). Contrary to this rather regular sinusoidal appearance, the averaged results (both with the technique of JLA and CC) for the remaining seven patients, with cortical myoclonic tremor, show minimal, if at all, short lived sinusoidal morphologies before and after time 0 on the averaged templates (Fig. 1a and c, Fig. 2c and d, Fig. 3a and b, and Fig. 4b, i and ii).

One of the patients (case 1) had no significant improvement after discontinuing treatment with lithium for 6 months. Case 2 noticed significant improvement of the myoclonus while cerebellar spectroscopy also improved (vermis NAA/Cr increased to 0.95 from 0.87, significant differences > 0.05) 6 months after adopting a gluten free diet. Another patient (case 4) showed no amelioration after her lithium daily dose was halved (from 800 to 400 mg). For the three patients referred from general neurology two with myoclonus, experienced mild disability and it was decided to follow them up without any changes of their psychoactive medication regime. The single patient with tremor was offered a beta blocker based on previous recommendations [32].

## Discussion

Lithium remains a widely used effective treatment for bipolar disorders [33] but its long-term use is associated with significant side effects, including renal, endocrine, dermatological and neurological manifestations [32]. Neurotoxicity can sometimes be irreversible and can occur after overdose or during maintenance treatment with lithium levels within the therapeutic range [34]. One of the most common side effects, reported in up to 25%, is tremor primarily affecting the hands [5]. Tremor commonly appears early on in the course of treatment and may improve over time. The tremor is reportedly indistinguishable from essential or enhanced physiologic tremor and patients typically do not have features of Parkinsonism [1]. Lithium induced tremor is more common in the elderly, possibly because of increased prevalence of essential tremor in this age group and, hence, the latter could be a predisposing factor. Furthermore, male gender, concurrent antidepressant (predominantly tricyclic antidepressants) and/or neuroleptic medications, higher doses of lithium with high serum levels, caffeine intake, alcohol withdrawal and anxiety are contributing factors [1, 3–5, 35–37] for the development of tremor.

The pathophysiological characteristics of lithium tremor remain poorly defined and is not yet clear if presentation in the early versus late phases of treatment exhibits distinguishing features. However, a coarse irregular hand tremor together with symptoms of ataxia, nystagmus, dysarthria and confusion is very suggestive of lithium toxicity [3, 38]. Lithium induced tremor may consist of rhythmical oscillations but it has also been described as an irregular, non-rhythmical tremor of variable intensity and frequency [6]. This later description renders clinical distinction between tremor and myoclonus problematic even if the latter is commonly characterised by an irregular, jerky appearance [39].

Myoclonus can be an adverse side effect of lithium monotherapy [11] or in combination with other drugs such as neuroleptics e.g. clozapine [40], and tricyclic antidepressants [41]. The presence of enlarged SEPs in patients receiving antidepressants in addition to lithium raises suspicion that the myoclonus is cortically driven [41]. This hypothesis was confirmed for a single case where the technique of JLA was applied in a patient where lithium with an antidepressant were prescribed [42].

A previous publication described five cases with cortical action myoclonus while on treatment with lithium (one also receiving antidepressant medications – sertraline and nefazodone) with no signs of neurotoxicity or epileptiform abnormalities on their routine EEGs [43]. Only one of the cases, on lithium monotherapy, showed enlarged SEPs.

Diffuse slow wave activity and epileptiform discharges are typical EEG findings of lithium toxicity [44]. However, no significant background slowing was seen in any of the EEGs of our patients with only occasional focal epileptiform discharges in one but no obvious association to the myoclonus. Overall, there was no significant electroencephalographic evidence to suggest lithium neurotoxicity in any of our cases, in keeping with the normal serum levels of the drug.

A synergistic effect of different factors appears to be contributing to the development of cortical myoclonus. Firstly, prolonged exposure to lithium (7 to 40 years). Secondly additional treatment with various types of antidepressant medications. Thirdly, 4 of the seven cases with myoclonus had gluten sensitivity, a factor linked to cerebellar ataxia and/or cortical myoclonus [21, 45–48]. Even allowing for the small size of our cohort, 57% had gluten sensitivity when compared to 11% in the general population [49]. Despite no evidence of lithium toxicity in any of our cases, six out of the seven patients with cortical myoclonus were female. Previous observations show that toxicity, that includes a variety of symptoms in addition to tremor, is more frequent amongst women [50, 51]. It is therefore possible that female gender may be another predisposing factor. Alcohol may also be another contributory factor perhaps through causing cerebellar degeneration as seen in two of our patients [52]. The underlying disease process may also be contributory: There is evidence of dysfunction on fMRI scans, involving timing networks, that include the cerebellar vermis in patients with psychosis [53], further supporting previous evidence of vermian atrophy in psychiatric patients [54]. In addition, it has been shown that patients with psychotic bipolar disorder demonstrate dysconnectivity of selective cerebro-cerebellar networks not attributed to the effect of medication or other substance use [55]. It is therefore possible that many of the psychiatric patients on treatment with lithium have an inherent cerebellar vulnerability that could be aggravated by chronic exposure to lithium.

The single patient with non-myoclonic tremor had a strong family history of tremor and received additional antipsychotic medication, both being predisposing factors for the development of tremor. Despite the patient having no features of parkinsonism his tremor was asymmetric, involving mainly the right side, and tended to persist at rest. Electrophysiology (Fig. 4) showed that this regular 6 Hz tremor had features of a centrally driven tremor that would fit within the spectrum of essential tremor.

In all five cases where MRS was performed there was evidence of cerebellar dysfunction whilst the electrophysiological findings were suggesting a cortical generator of the myoclonic tremor. This is very much in keeping with pathological findings from other conditions where a phenotype of cortical myoclonic tremor is associated with cerebellar dysfunction, namely familial cortical tremor with epilepsy (FCTE) and progressive myoclonus ataxia (PMA) in coeliac disease [27, 48, 56–59]. Autopsy results on families affected by FCTE and on individual cases with PMA, shows that pathology is localising to the cerebellum [48, 56–58]. While the cortex appears intact, the electrophysiological findings show consistently a cortical generator while the SEPs are typically “Giant” with or without evidence of stimulus sensitive myoclonus (i.e. cortical reflexes, “c-reflexes”). It is postulated that reduced inhibition of the motor cortex due to dysfunctional cerebellar output results in cortical hyperexcitability [57], however, only one of our seven patients with cortical myoclonus had giant SEPs.

Despite the fact that lithium treatment was stopped in one of our patients and halved in another, this did not translate into clinical improvement of their myoclonus. This very limited evidence suggests that cortical myoclonus may be an irreversible adverse effect of lithium when there is superimposed pathology affecting the cerebellum. Only one patient experienced improvement of her myoclonus on a gluten free diet, without any changes on the lithium daily dose. The same patient showed improvement of cerebellar functioning on MRS. Overall the myoclonic tremor encountered in these patients is debilitating and difficult to treat. We have used levetiracetam successfully in cases of cortical myoclonus due to other aetiologies but we have been reluctant to use this drug in this group of patients with susceptibility to depression and behavioural issues. Furthermore, most of these patients were already on many psychotropic drugs.

## Conclusions

We provide electrophysiological evidence of cortical myoclonus in seven patients presenting with an upper limb tremor after long-term exposure to lithium. We identified cerebellar dysfunction as being very prevalent in this cohort. We postulate that factors affecting cerebellar function are crucial for the emergence of myoclonus in the context of prolonged lithium exposure. Without appropriate polygraphy recordings, differentiating between lithium induced tremor and cortical myoclonus is not possible. Lithium induced cortical myoclonus may not be as rare as the paucity of published cases suggests.

## Additional file

Additional file 1: “Cortico-muscular latencies” for 7 cases shown in **Figures S1**, **S2**, **S3** and **S4.** (PDF 2500 kb)

## Abbreviations

CC: Cross-correlation
FCTE: Familial cortical tremor with epilepsy
FFT: Fast Fourier transform
JLA: Jerk-locked averaging
MRS: Magnetic resonance spectroscopy
NAA/Cr: N-acetyl-aspartate/creatine
NaSSA: Noradrenergic and specific serotonergic antidepressant
PDS: Paroxysmal depolarisation shift
PMA: Progressive myoclonus ataxia
SEP: Somatosensory evoked potentials
SNRI: Serotonin-norepinephrine reuptake inhibitor
SSRIs: Selective serotonin reuptake inhibitors
TCA: Tricyclic antidepressants

## References

[1] Baek JH, Kinrys G, Nierenberg AA (2014). Lithium tremor revisited: pathophysiology and treatment. Acta Psychiatr Scand.

[2] Canning JE, Burton S, Hall B (2012). Lithium and valproate-induced tremors. Mental Health Clin..

[3] Carroll JA, Jefferson JW, Greist JH (1987). Treating tremor induced by lithium. Hosp Community Psychiatry.

[4] Gelenberg AJ (1988). Lithium efficacy and adverse effects. J Clin Psychiatry..

[5] Gelenberg AJ, Jefferson JW (1995). Lithium tremor. J Clin Psychiatry.

[6] Lapierre YD (1976). Control of lithium tremor with propranolol. Can Med Assoc J.

[7] Netto I, Phutane VH. Reversible lithium neurotoxicity: review of the literatur. Prim Care Companion CNS Disord. 2012;14(1). 10.4088/PCC.11r01197. 10.4088/PCC.11r01197 PMC3357580 22690368

[8] Prettyman R (1994). Lithium neurotoxicity at subtherapeutic serum levels. Br J Psychiatry.

[9] Pullinger S, Tyrer P (1983). Acute lithium-induced tremor. Br J Psychiatry.

[10] Tyrer SP (1978). Lithium toxicity. Br Med J.

[11] Kores B, Lader MH (1997). Irreversible lithium neurotoxicity: an overview. Clin Neuropharmacol.

[12] Bhidayasiri R (2005). Differential diagnosis of common tremor syndromes. Postgrad Med J.

[13] Shibasaki H, Hallett M (2005). Electrophysiological studies of myoclonus. Muscle Nerve.

[14] Toro C, Pascual-Leone A, Deuschl G, Tate E, Pranzatelli MR, Hallett M (1993). Cortical tremor. A common manifestation of cortical myoclonus. Neurology..

[15] Caviness JN, Brown P (2004). Myoclonus: current concepts and recent advances. Lancet Neurol.

[16] Kojovic M, Cordivari C, Bhatia K (2011). Myoclonic disorders: a practical approach for diagnosis and treatment. Ther Adv Neurol Disord.

[17] Hallett M, Chadwick D, Marsden CD (1979). Cortical reflex myoclonus. Neurology..

[18] Mima T, Nagamine T, Ikeda A, Yazawa S, Kimura J, Shibasaki H (1998). Pathogenesis of cortical myoclonus studied by magnetoencephalography. Ann Neurol.

[19] Currie S, Hadjivassiliou M, Craven IJ, Wilkinson ID, Griffiths PD, Hoggard N (2013). Magnetic resonance spectroscopy of the brain. Postgrad Med J.

[20] Baldarcara L, Currie S, Hadjivassiliou M, Hoggard N, Jack A, Jackowski AP (2015). Consensus paper: radiological biomarkers of cerebellar diseases. Cerebellum..

[21] Hadjivassiliou M, Grunewald RA, Sanders DS, Shanmugarajah P, Hoggard N (2017). Effect of gluten-free diet on cerebellar MR spectroscopy in gluten ataxia. Neurology..

[22] Hadjivassiliou M, Wallis LI, Hoggard N, Grunewald RA, Griffiths PD, Wilkinson ID (2012). MR spectroscopy and atrophy in gluten, Friedreich's and SCA6 ataxias. Acta Neurol Scand.

[23] Deuschl G, Raethjen J, Lindemann M, Krack P (2001). The pathophysiology of tremor. Muscle Nerve.

[24] Grosse P, Guerrini R, Parmeggiani L, Bonanni P, Pogosyan A, Brown P (2003). Abnormal corticomuscular and intermuscular coupling in high-frequency rhythmic myoclonus. Brain..

[25] Brown P, Farmer SF, Halliday DM, Marsden J, Rosenberg JR (1999). Coherent cortical and muscle discharge in cortical myoclonus. Brain..

[26] Obeso JA, Rothwell JC, Marsden CD (1985). The spectrum of cortical myoclonus. From focal reflex jerks to spontaneous motor epilepsy. Brain..

[27] Shibasaki H. Cortical activities associated with voluntary movements and involuntary movements. Clin Neurophysiol. 2012;123(2):229-43. 10.1016/j.clinph.2011.07.042. 10.1016/j.clinph.2011.07.042 21906995

[28] Shibasaki H, Shima F, Kuroiwa Y (1978). Clinical studies of the movement-related cortical potential (MP) and the relationship between the dentatorubrothalamic pathway and readiness potential (RP). J Neurol.

[29] Barlow JS (1959). Autocorrelation and Crosscorrelation analysis in electroencephalography. IRE Trans Med Electron.

[30] Cruccu G, Aminoff MJ, Curio G, Guerit JM, Kakigi R, Mauguiere F (2008). Recommendations for the clinical use of somatosensory-evoked potentials. Clin Neurophysiol.

[31] Cassim F, Houdayer E (2006). Neurophysiology of myoclonus. Neurophysiol Clin.

[32] Gitlin M (2016). Lithium side effects and toxicity: prevalence and management strategies. Int J Bipolar Disord.

[33] DeJongh B (2012). Lithium use in bipolar disorder: summary of evidence for acute mania, acute depression, and maintenance treatment. Mental Health Clin.

[34] Shah VC, Kayathi P, Singh G, Lippmann S. Enhance your understanding of Lithium neurotoxicity. Prim Care Companion CNS Disord. 2015;17(3). 10.4088/PCC.14l01767. 10.4088/PCC.14l01767 PMC4578904 26644952

[35] Gelenberg AJ, Kane JM, Keller MB, Lavori P, Rosenbaum JF, Cole K (1989). Comparison of standard and low serum levels of lithium for maintenance treatment of bipolar disorder. N Engl J Med.

[36] Sengupta M, Guha P, Banerjee P, Paul S, Baisya R, Bhattacharya K. Comparison of tremor induced by valproate and lithium in bipolar disorder using a hand steadiness tester. Int J Basic Clin Pharmacol. 2014;3(1):151-154.

[37] Carney SM, Goodwin GM. Lithium - a continuing story in the treatment of bipolar disorder. Acta Psychiatr Scand Suppl. 2005;(426):7–12. 10.1111/j.1600-0447.2005.00521.x 15833095

[38] Ivkovic A, Stern TA. Lithium-Induced Neurotoxicity: Clinical Presentations, Pathophysiology, and Treatment. Psychosomatics. 2014;55(3):296-302. 10.1016/j.psym.2013.11.007 24388123

[39] Termsarasab P, Frucht SJ (2015). Cortical tremor (CT) with coincident orthostatic movements. J Clin Mov Disord.

[40] Lemus CZ, Lieberman JA, Johns CA (1989). Myoclonus during treatment with clozapine and lithium: the role of serotonin. Hillside J Clin Psychiatry.

[41] Forstl H, Pohlmann-Eden B (1990). Amplitudes of somatosensory evoked potentials reflect cortical hyperexcitability in antidepressant-induced myoclonus. Neurology..

[42] Evidente VG, Caviness JN (1999). Focal cortical transient preceding myoclonus during lithium and tricyclic antidepressant therapy. Neurology..

[43] Caviness JN, Evidente VG (2003). Cortical myoclonus during lithium exposure. Arch Neurol.

[44] Hansen HE, Amdisen A (1978). Lithium intoxication. (report of 23 cases and review of 100 cases from the literature). Q J Med.

[45] Hadjivassiliou M, Martindale J, Shanmugarajah P, Grünewald RA, Sarrigiannis PG, Beauchamp N (2017). Causes of progressive cerebellar ataxia: prospective evaluation of 1500 patients. J Neurol Neurosurg Psychiatry.

[46] Hadjivassiliou M, Grünewald R, Sharrack B, Sanders D, Lobo A, Williamson C (2003). Gluten ataxia in perspective: epidemiology, genetic susceptibility and clinical characteristics. Brain..

[47] Hadjivassiliou M, Sanders DS, Grünewald RA, Woodroofe N, Boscolo S, Aeschlimann D (2010). Gluten sensitivity: from gut to brain. Lancet Neurol.

[48] Sarrigiannis PG, Hoggard N, Aeschlimann D, Sanders DS, Grunewald RA, Unwin ZC (2014). Myoclonus ataxia and refractory coeliac disease. Cerebellum Ataxias.

[49] Sanders DS, Patel D, Stephenson TJ, Ward AM, McCloskey EV, Hadjivassiliou M (2003). A primary care cross-sectional study of undiagnosed adult coeliac disease. Eur J Gastroenterol Hepatol.

[50] Schou M (1984). Long-lasting neurological sequelae after lithium intoxication. Acta Psychiatr Scand.

[51] Donaldson IM, Cuningham J (1983). Persisting neurologic sequelae of lithium carbonate therapy. Arch Neurol.

[52] Verdoux H, Bourgeois ML (1990). A case of lithium neurotoxicity with irreversible cerebellar syndrome. J Nerv Ment Dis.

[53] Losak J, Huttlova J, Lipova P, Marecek R, Bares M, Filip P (2016). Predictive motor timing and the cerebellar vermis in schizophrenia: an fMRI study. Schizophr Bull.

[54] Heath RG, Franklin DE, Walker CF, Keating JW (1982). Cerebellar vermal atrophy in psychiatric patients. Biol Psychiatry.

[55] Shinn AK, Roh YS, Ravichandran CT, Baker JT, Ongur D, Cohen BM (2017). Aberrant cerebellar connectivity in bipolar disorder with psychosis. Biol Psychiatry Cogn Neurosci Neuroimaging.

[56] Bhatia KP, Brown P, Gregory R, Lennox GG, Manji H, Thompson PD (1995). Progressive myoclonic ataxia associated with coeliac disease. The myoclonus is of cortical origin, but the pathology is in the cerebellum. Brain..

[57] Tijssen MA, Thom M, Ellison DW, Wilkins P, Barnes D, Thompson PD (2000). Cortical myoclonus and cerebellar pathology. Neurology..

[58] van Rootselaar AF, Aronica E, Jansen Steur EN, Rozemuller-Kwakkel JM, de Vos RA, Tijssen MA (2004). Familial cortical tremor with epilepsy and cerebellar pathological findings. Mov Disord.

[59] van Rootselaar AF, van Schaik IN, van den Maagdenberg AM, Koelman JH, Callenbach PM, Tijssen MA (2005). Familial cortical myoclonic tremor with epilepsy: a single syndromic classification for a group of pedigrees bearing common features. Mov Disord.

