# Supplementary material for: Tremor after long term lithium treatment; is it cortical myoclonus?
Source: Cerebellum Ataxias. 2019 May 22;6:5. doi: 10.1186/s40673-019-0100-y (PMC6532190; doi:10.1186/s40673-019-0100-y)

Cortico-muscular latencies for JLA and CC shown in figures 1,2,3 &4

Analysis performed in Spike 2 (Version 9.04) software

Case 1 Figure 1,B

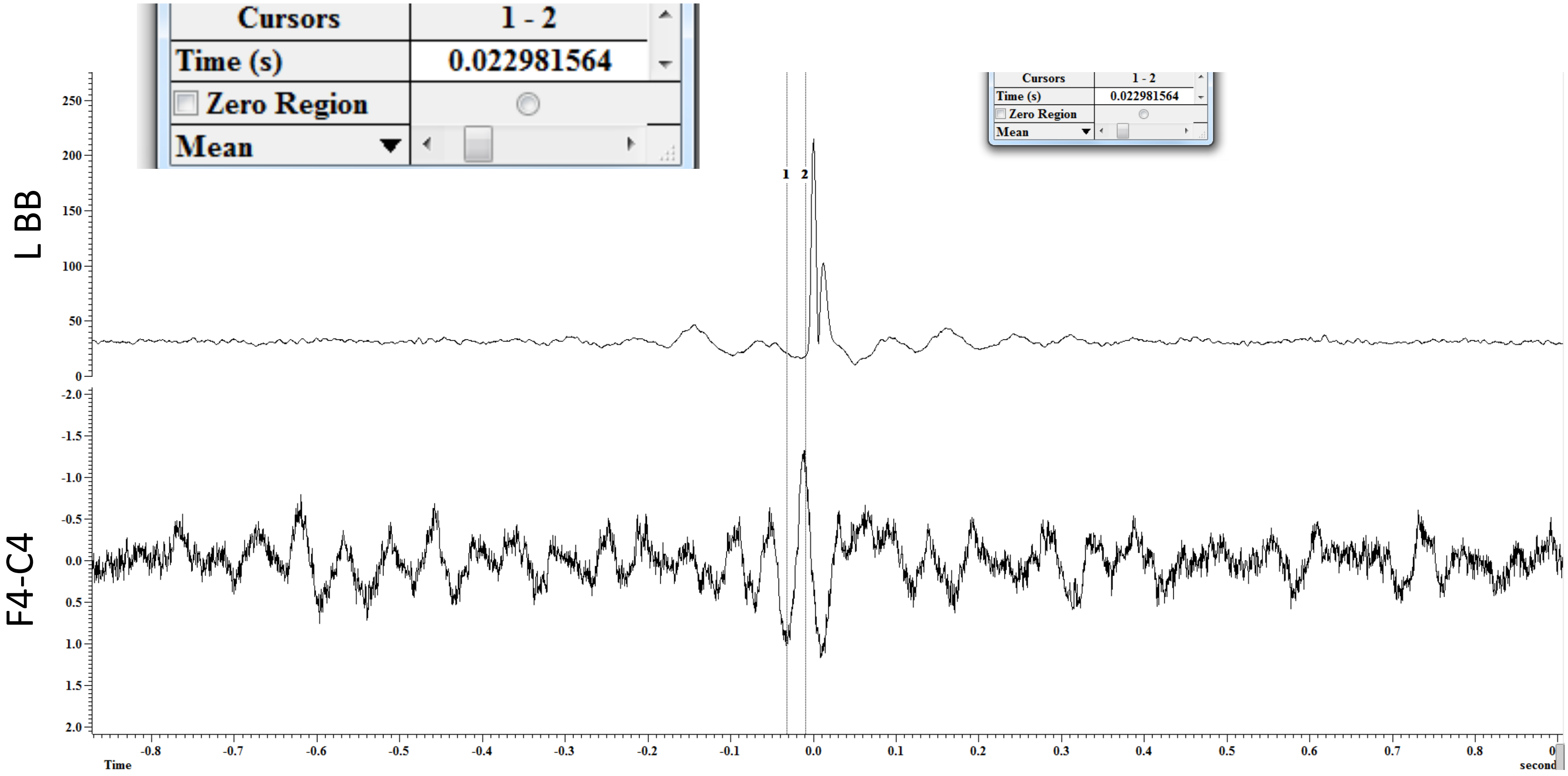

Case 2 figure 2,A

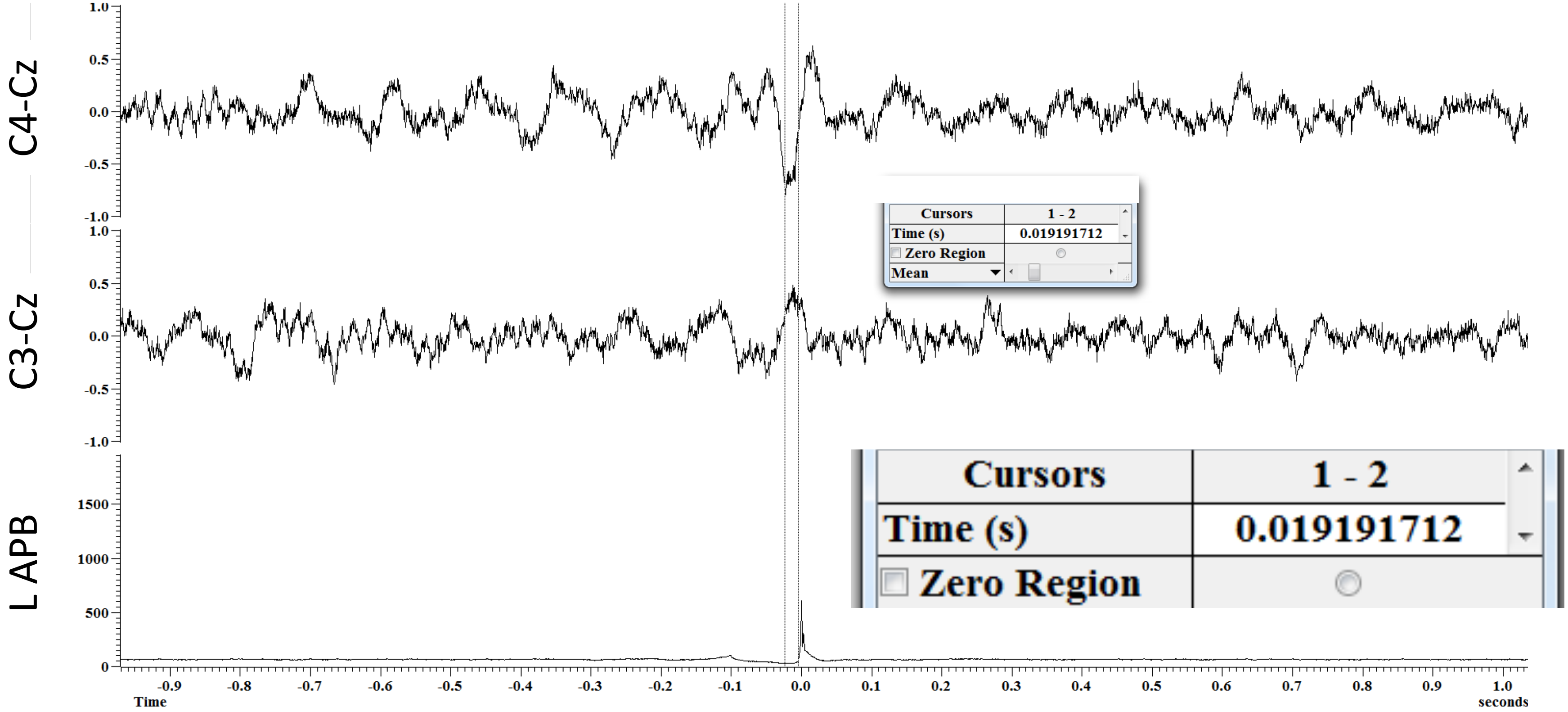

Case 3 figure 2,B

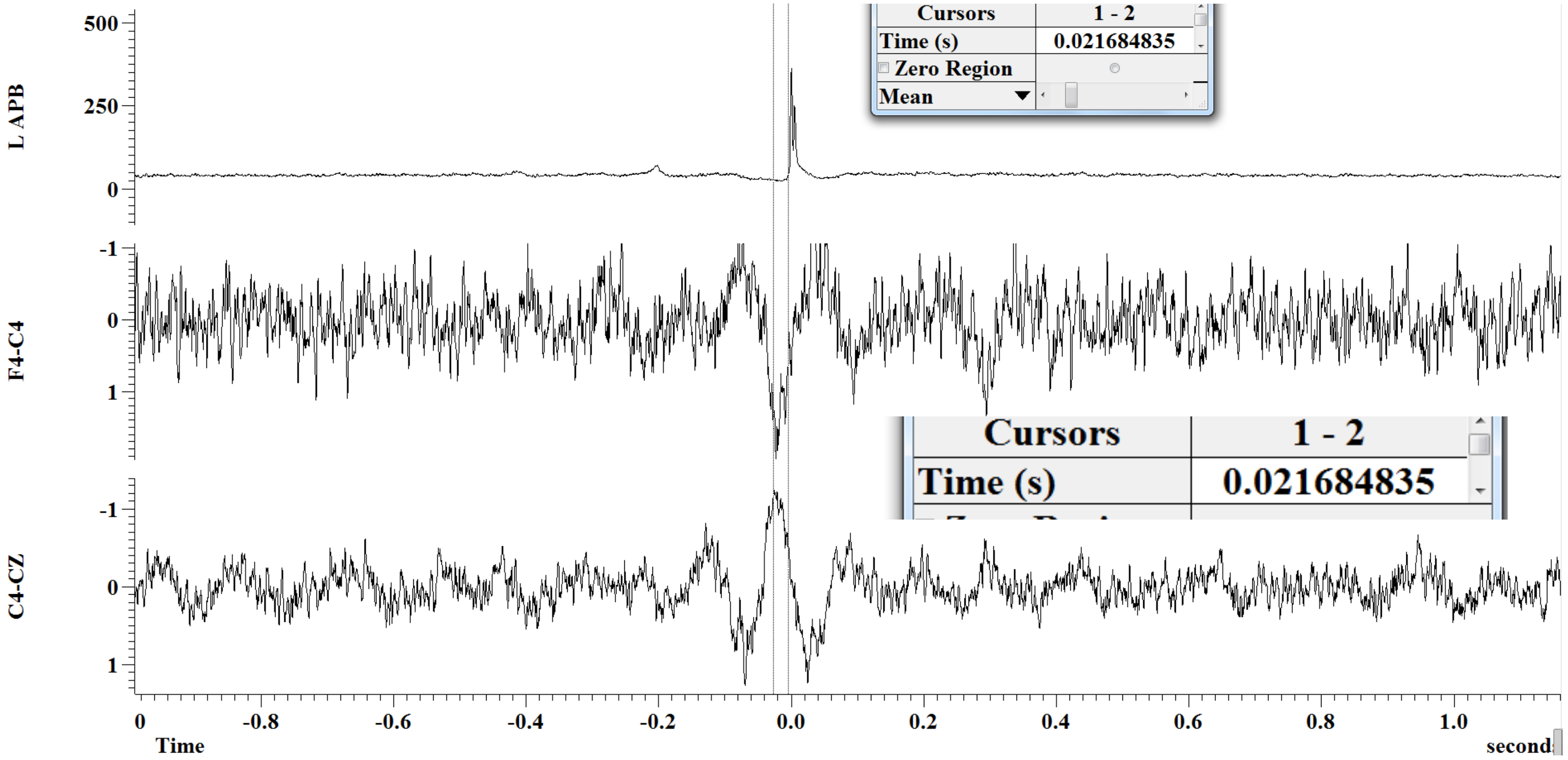

Case 4 Figure 2,C

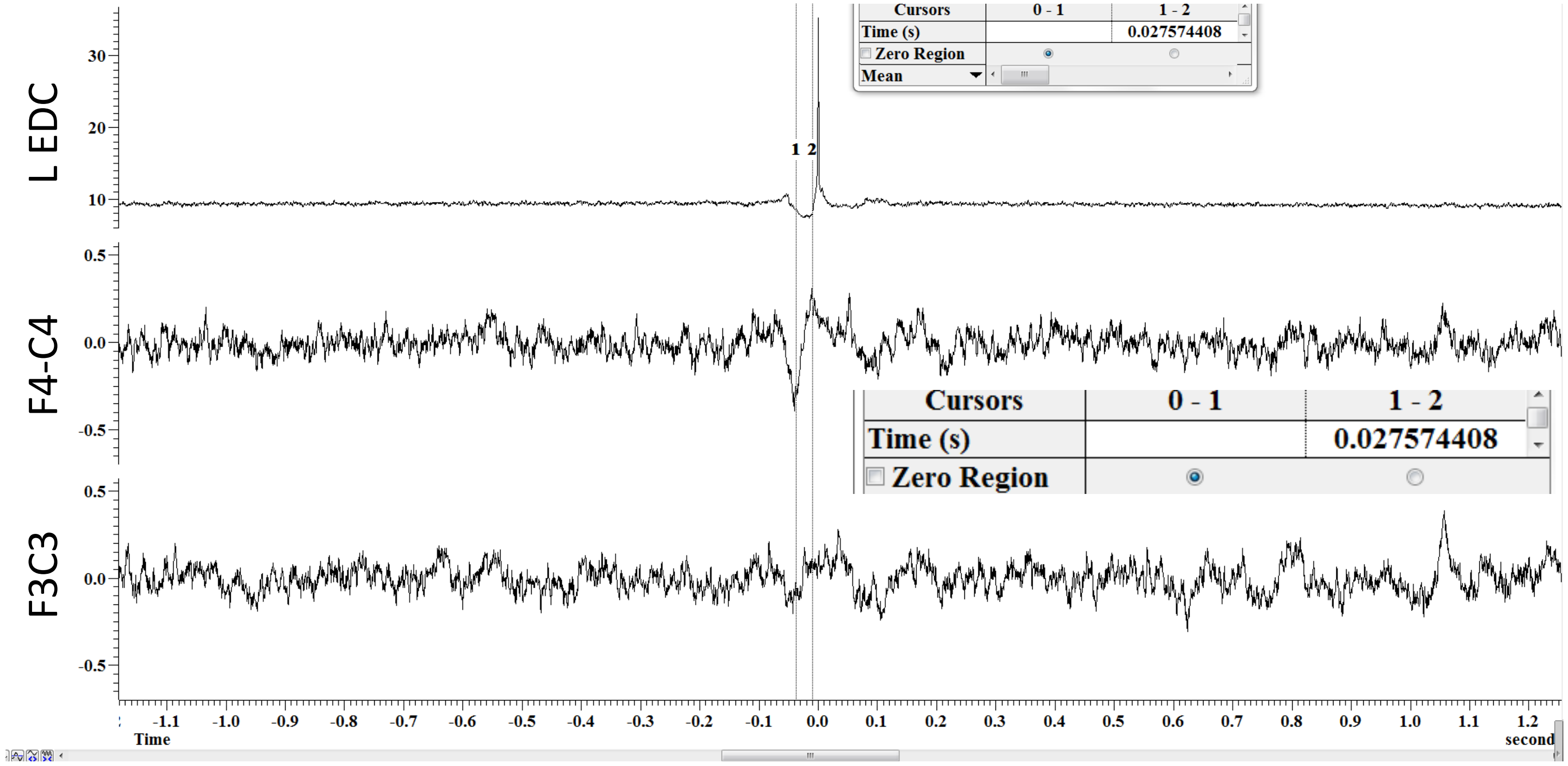

Case 5 Figure 2,D

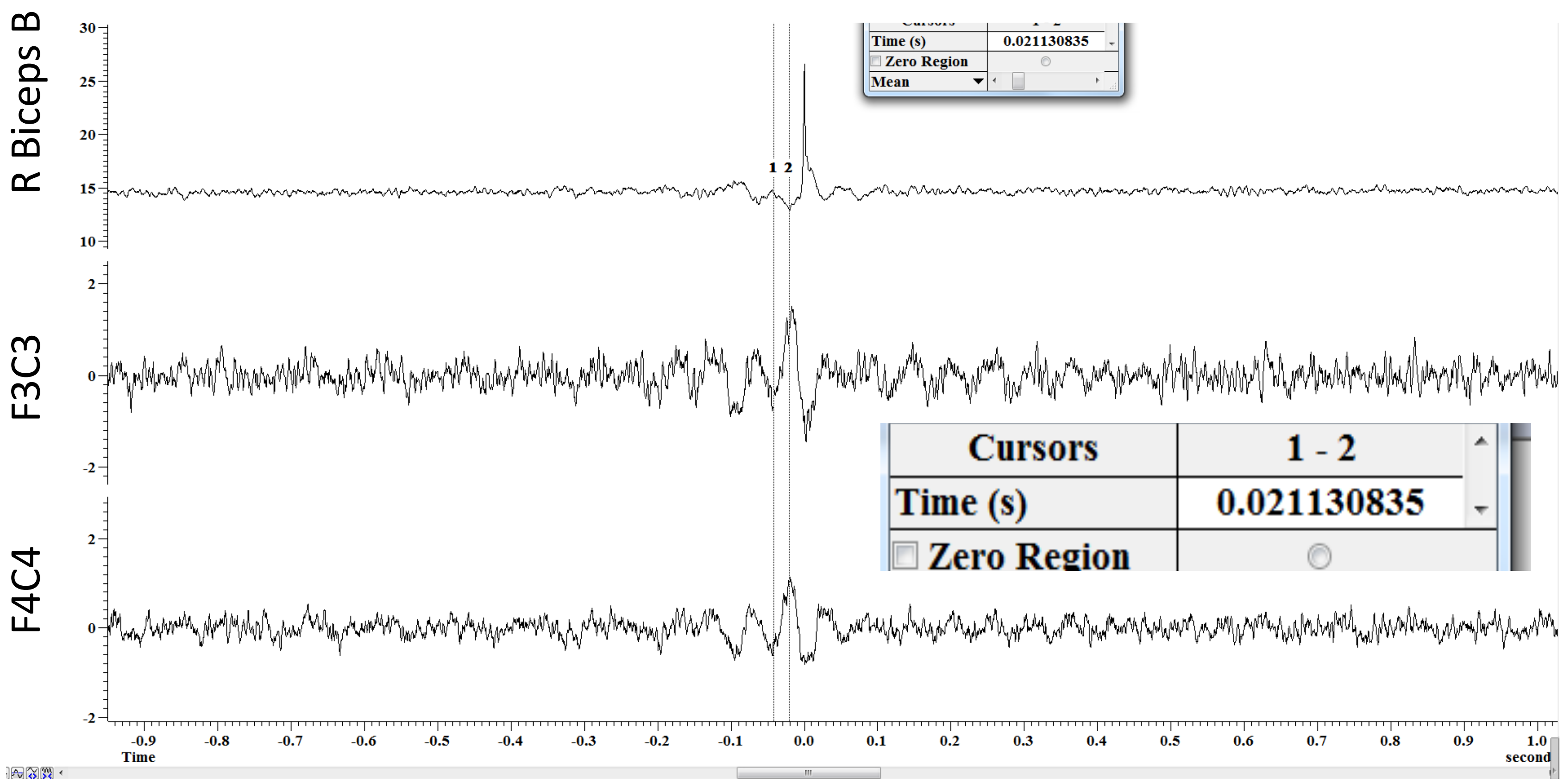

Case 6 Figure 3,B

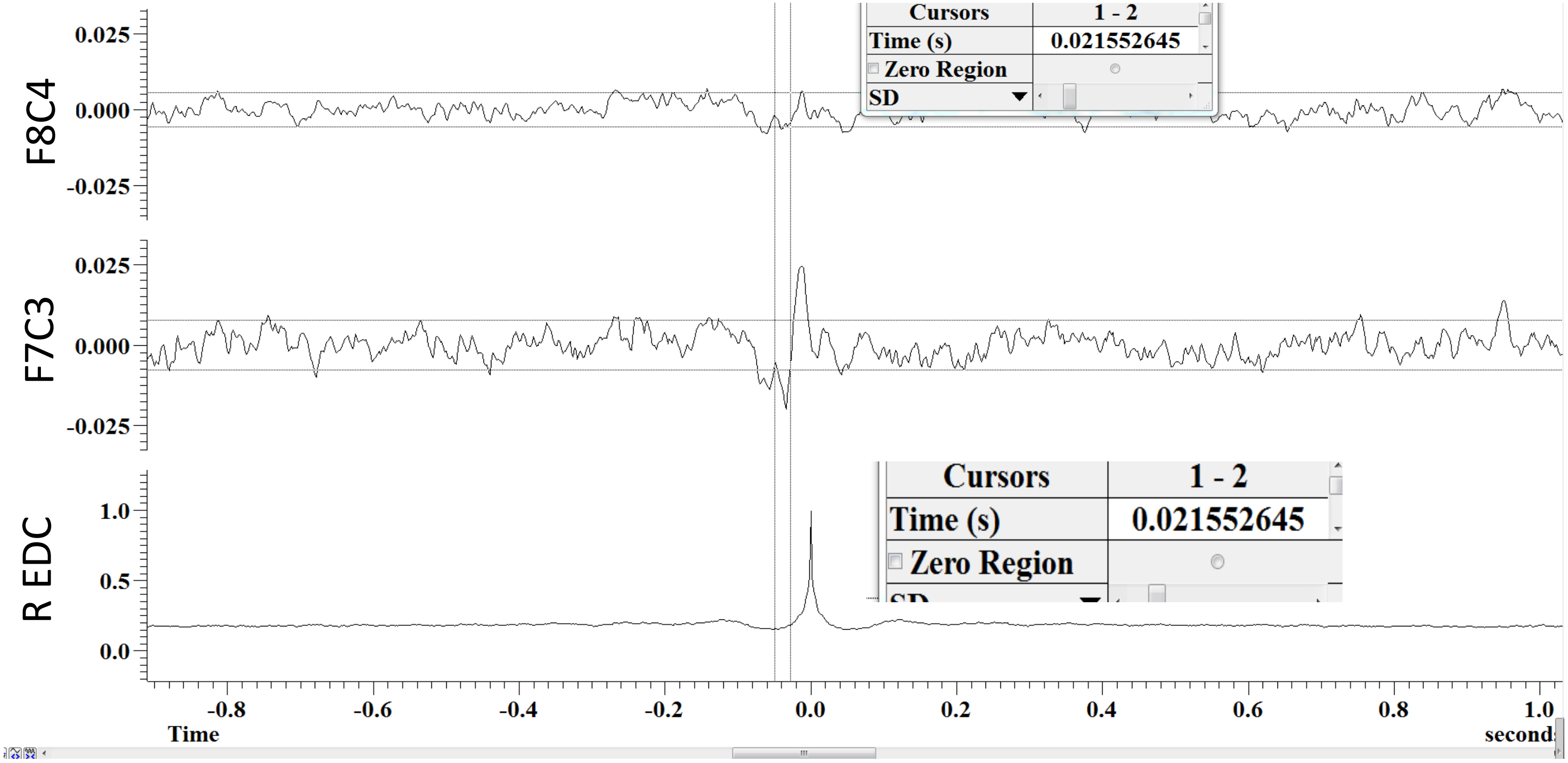

Case 7 Figure 4,

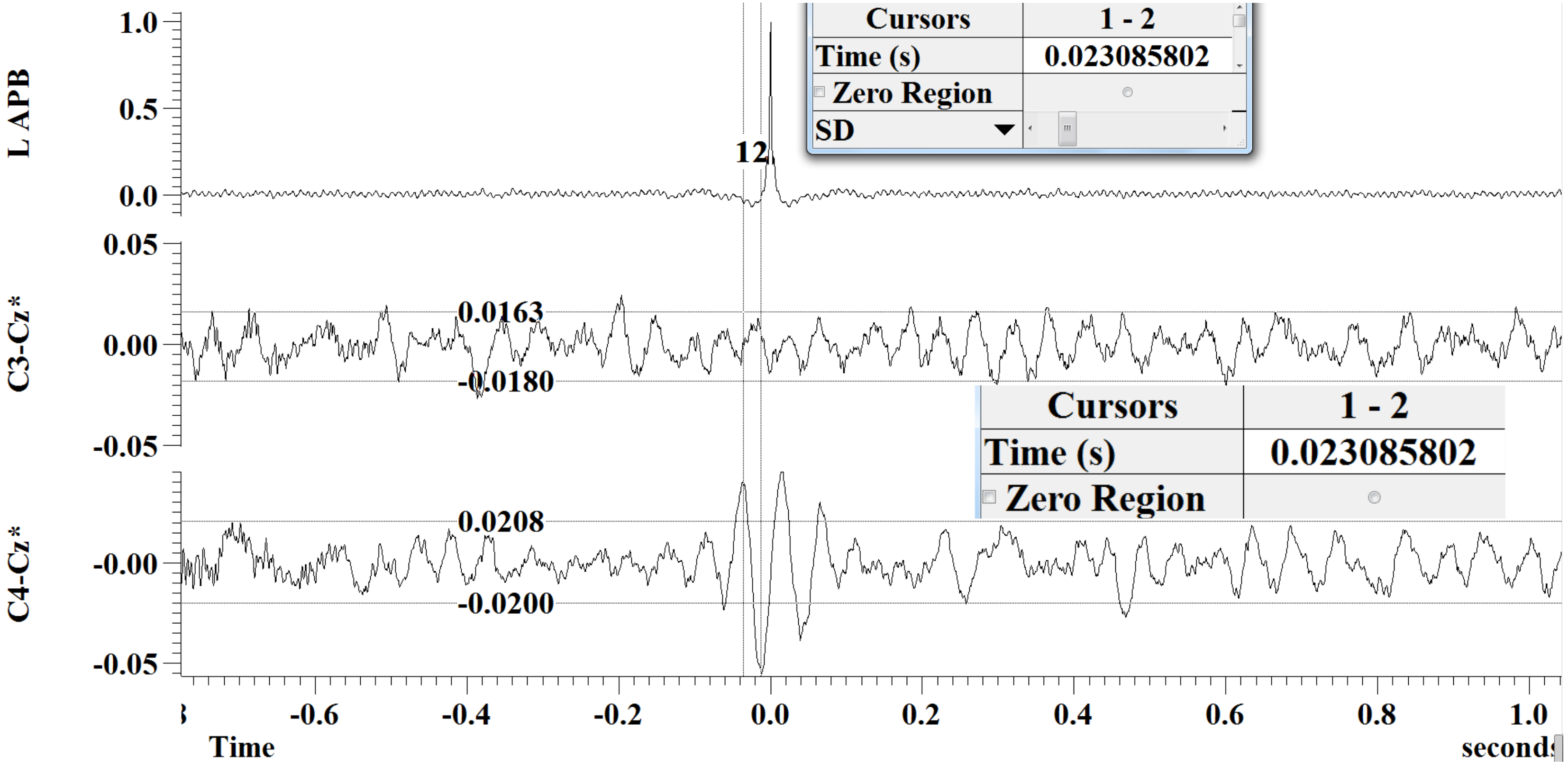

Supplement: Supplementary file 1 — “Cortico-muscular latencies” for 7 cases shown in Figures S1, S2, S3 and S4. (PDF 2500 kb) [file 40673_2019_100_MOESM1_ESM.pdf]
